# Carvacrol and *p*-cymene inactivate *Escherichia coli *O157:H7 in apple juice

**DOI:** 10.1186/1471-2180-5-36

**Published:** 2005-06-17

**Authors:** Gabriella Kiskó, Sibel Roller

**Affiliations:** 1 Corvinus University of Budapest, 1118 Budapest, Hungary; 2 Microbiology Research Group, Faculty of Health and Human Sciences, Thames Valley University, 18–22 Bond Street, LondonW5 5AA, UK

## Abstract

**Background:**

Outbreaks of food poisoning associated with drinking un-pasteurised apple juice contaminated with enterohaemorrhagic *Escherichia coli *O157:H7 are a cause of serious illness and occasionally death. Whilst a well-established heat process (pasteurisation) will readily eliminate the pathogen, some consumers are demanding more fresh-like foods that have not been subjected to processing methods that are perceived as severe and may lead to loss of flavour and vitamins. Therefore, alternative methods are being investigated to replace pasteurisation and improve the safety of minimally-processed juices. The addition of natural antimicrobial substances such as the phenolic substances carvacrol and *p-*cymene (derived from the essential oils of herbs and spices) provides a potential new route to assure safety and extend the shelf-life of raw fruit juices.

The aim of this study was to evaluate the addition of very low concentrations (0.25–1.25 mM) of carvacrol and *p-*cymene both individually and in combination as a novel means of controlling *Escherichia coli *O157:H7 in un-pasteurised apple juice.

**Results:**

When inoculated at a level of 4 log CFU/ml into un-pasteurised apple juice (pH 3.20 ± 0.06), *Escherichia coli *O157:H7 survived for up to 3 and 19 days at 25° and 4°C, respectively. Treatment of the juice with 1.25 mM carvacrol or *p-*cymene reduced the numbers of *E. coli *O157:H7 to undetectable levels within 1–2 days at both storage temperatures. The effective concentrations of carvacrol could be reduced even further by combining it at 0.5 mM with cymene at 0.25 mM. The phenolic compounds were biocidal against both spoilage yeasts and *E. coli *O157:H7 thereby increasing the shelf-life and improving the safety of un-pasteurised apple juice, particularly when stored at chill temperatures.

**Conclusion:**

The results showed that the natural antimicrobial compounds carvacrol and p-cymene could potentially be used to extend the shelf life and improve the safety margins in un-pasteurised chilled fruit juices.

## Background

Conventional fruit juice processing involves a heating step to inactivate the vegetative forms of pathogenic and spoilage microorganisms. Any remaining bacterial spores are generally unable to germinate due to the acidic nature of the juices [1]. This ensures acceptable safety margins and extends the shelf-life of the juice. However, heat treatment causes vitamin losses and changes in flavour of the juices and some consumers regard heat-treated, shelf-stable products as low in quality. In the last 15 years, there have been several outbreaks of food poisoning associated with drinking un-pasteurised apple juice contaminated with enterohaemorrhagic *Escherichia coli *O157:H7 and several children have died in the USA [2]. Warning labels are now required in the USA for all fruit juices unless a 5-log pathogen reduction treatment has been applied to the product [3-5]. Therefore, there is a need for new methods of fruit juice preservation that rely less heavily on severe heat treatment or the addition of synthetic preservatives. It has been suggested that many natural antimicrobial compounds from plant, animal and microbial sources might fulfil this need [6-8].

Carvacrol (2-methyl-5-(1-methylethyl)-phenol) is a major component of the essential oils of oregano and thyme [9,10]. Generally recognized as a safe food additive, carvacrol is used as a flavouring agent in baked goods, sweets, beverages and chewing gum [11]. Carvacrol-containing essential oils are biostatic and/or biocidal against many bacteria, yeast and fungi in laboratory media and consequently have attracted considerable research attention as potential food preservatives [reviewed in [12]]. Carvacrol has also been shown to inactivate microorganisms in biofilms on stainless steel surfaces [13,14]. The biocidal mode of action of carvacrol on bacteria is similar to that of other phenolic compounds and occurs *via *membrane damage resulting in an increase in membrane permeability to protons and potassium ions, depletion of the intracellular ATP pool and disruption of the proton-motive force [15,16].

The biological precursor of carvacrol, *p-*cymene (1-methyl-4-(1-methylethyl)-benzene), is less antimicrobial than carvacrol when used alone. *p-*Cymene lacks a hydroxyl group, which is thought to play an important role in antimicrobial activity [17]. Synergism between carvacrol and *p-*cymene against *B. cereus in vitro *and in rice has been reported [18].

The minimum inhibitory concentrations (MICs) of carvacrol, thymol, cinnamic acid and other phenolic compounds from herbs and spices against some food-borne bacteria *in vitro *have been reported at around 1 mM [12,18,19]. However, a much higher concentration is usually needed to achieve the same biocidal or biostatic effects in foods. For example, the MIC of carvacrol in mushroom soup inoculated with *B. cereus *was 50 times higher than that found in laboratory media [20]. Similarly, the concentrations of carvacrol and cinnamic acid required to delay spoilage of mango, kiwifruit and melon slices was >5 mM [21,22]. A number of intrinsic (fat and protein content, pH, salt, etc.) and extrinsic (temperature, oxygen availability, etc.) factors are thought to play a role in protecting microorganisms from the biocidal effects of carvacrol in foods. Furthermore, the potent aromatic and antioxidant properties of phenolic compounds at these high concentrations have been reported to lead to undesirable colour, odour and flavour changes in food products [8,22].

The aim of this study was to evaluate the addition of very low concentrations of carvacrol and *p-*cymene both individually and in combination as a novel means of controlling *Escherichia coli *O157:H7 in un-pasteurised apple juice.

## Results

### Inactivation/inhibition of microorganisms with 1.25 mM carvacrol and *p-*cymene used individually

**Figure 1:**
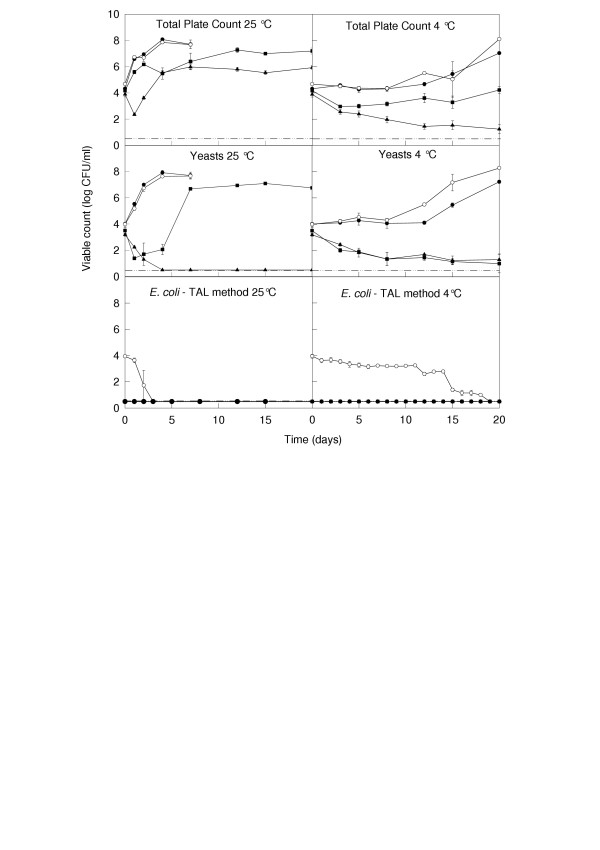
**Survival/growth of microorganisms in apple juice treated with 1.25 mM carvacrol or *p-*cymene at 25° and 4°C. **Data points represent: control with no additions (●); control inoculated with *E. coli *O157:H7 (○); juice inoculated with *E. coli *O157:H7 and treated with 1.25 mM carvacrol (▲); juice inoculated with *E. coli *O157:H7 and treated with 1.25 mM *p-*cymene (■). Sampling of control batches at 25°C was discontinued after 7 d due tovisible spoilage. Dotted line represents the lower detection limit of the plating technique. The pH of the juice was 3.17 on Day 0 and ranged between 3.30 and 3.44 on the final day of sampling (Days 7 and 20 at 4° and 25°C, respectively).

At 25°C, the total viable counts in un-pasteurized, untreated apple juice increased in the first 4 days to a level of 8 log CFU/ml, where they remained up to Day 7 (Figure 1). Yeast counts reflected the total counts. When inoculated into raw apple juice, *E. coli *O157:H7 survived for up to 3 days at 25°C but by Day 4, numbers decreased to below the detection limit of the plate-counting method (0.5 log CFU/ml). Enrichment methods allowed the detection of *E coli *up to 3 days after inoculation at 25°C but by Day 4 (and beyond to Day 20) none were detected (Table 1).

In the presence of 1.25 mM carvacrol, there was an initial reduction (within the first two days) in total and yeast counts of about 2 log CFU/ml (Figure 1). This was followed by re-growth of total viable numbers to about 6 log CFU/ml within 7 days where they remained steady until Day 20. Yeast numbers continued to fall in the presence of 1.25 mM carvacrol and by Day 4 reached levels below the detection limit of the assay (0.5 log CFU/ml). In the presence of 1.25 mM *p-*cymene, total plate counts increased more slowly than in the untreated controls but by Day 12, numbers were similar to those in the controls. Yeasts were initially inactivated by *p-*cymene and remained at low levels (1–2 log CFU/ml) from Day 1 to 4 but then increased to about 6–7 log CFU/ml by Day 8 where they remained up to Day 20. Notably, numbers of *E. coli *decreased to below the limit of detection of the plate-counting method within less than one day of exposure to both carvacrol and p-cymene. *E. coli *were no longer detectable by enrichment methods after 2 days in the presence of carvacrol and after 3 days in the presence of *p-*cymene (Table 1).

At 4°C, total viable numbers and yeasts in the untreated juice remained at about 4–5 log CFU/ml for the first 12 days of incubation, followed by very slow growth to about 7 log CFU/ml by Day 20 (Figure 1). In the presence of 1.25 mM carvacrol and p-cymene, there was a gradual decline in total and yeast numbers with carvacrol affecting a slightly steeper reduction in total viable numbers than *p-*cymene. By Day 20, total numbers were about 1 and 4 log CFU/ml in the presence of 1.25 mM carvacrol and p-cymene, respectively. Notably, *E. coli *O157:H7 survived at a level of 3–4 log CFU/ml for up to 14 days at 4°C, was still countable on Day 18 (1 log CFU/ml) and was detectable (by enrichment) on Day 19 of incubation. In contrast, *E coli *was not countable (above the detection limit of 0.5 log CFU/ml) or detectable (by enrichment) after 1 day of exposure to either carvacrol or p-cymene (Figure 1). The results for *E. coli *shown in Figure 1 were obtained using thin TSA overlaid on selective CT-SMAC agar (see Methods section) in an attempt to resuscitate injured organisms. The results obtained on plain CT-SMAC agar and on Chromagar (with and without a thin agar layer on top) were very similar and are consequently not illustrated.

It was noted that the addition of either carvacrol or cymene at 1.25 mM imparted an intense "spicy" aroma to the treated apple juices.

### Inactivation of microorganisms with 0.5 mM carvacrol and/or 0.25 mM *p-*cymene at 4°C

**Figure 2:**
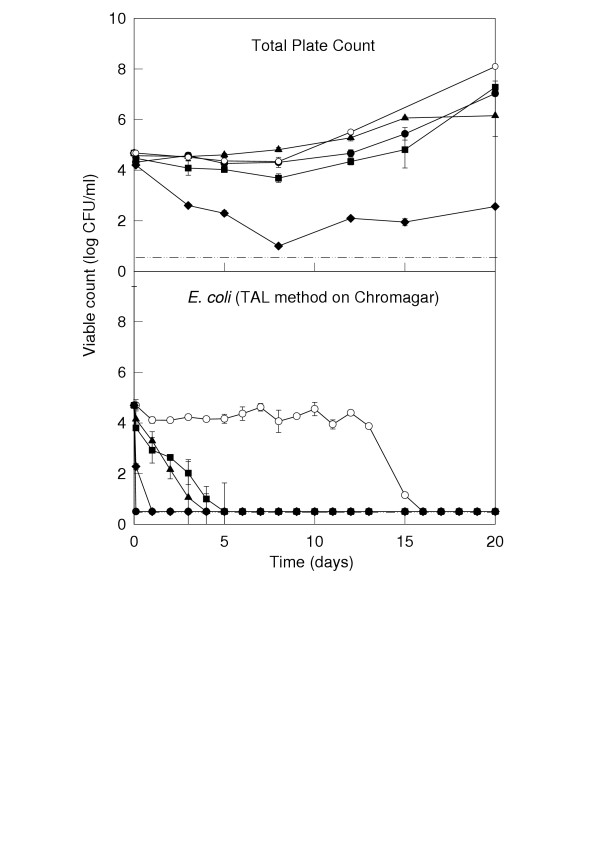
**Survival of microorganisms in apple juice treated with 0.5 mM carvacrol and/or 0.25 mM *p-*cymene at 4°C. **Data points represent: control with no additions (●); control inoculated with *E. coli *O157:H7 (○); juice inoculated with *E. coli *O157:H7 and treated with 0.5 mM carvacrol (▲); juice inoculated with *E. coli *O157:H7 and treated with 0.25 mM cymene (■); and juice inoculated with *E. coli *O157:H7 and treated with the combination of 0.5 mM carvacrol plus 0.25 mM cymene (◆). The dotted line represents the lower detection limit of the plating technique. The pH of the juice was 3.21 on Day 0 and ranged between 3.16 and 3.32 on the final day of sampling (Day 20).

Since the results above indicated that survival of *E. coli *O157:H7 was substantially extended at chill temperatures and therefore represented a greater cause for concern than survival at ambient temperatures, further work was undertaken at 4°C only. In order to study the effect of combinations of carvacrol and cymene, both compounds were used at concentrations that had no or very little inhibitory effect on microorganisms. As shown in Figure 2, addition of 0.5 mM carvacrol or 0.25 mM *p-*cymene individually to apple juice had a very slight inhibitory effect on total microbial counts at 4°C. Like in Figure 1, survival of *E. coli *O157:H7 persisted for two weeks at this temperature (Figure 2 and Table 2). Using the plate-counting technique, it is shown in Figure 2 that survival of *E. coli *was curtailed by both 0.5 mM carvacrol and 0.25 mM p-cymene to about 5 days. Enrichment techniques showed that *E. coli *was detectable up to 7 and 14 days in the presence of 0.5 and 0.25 mM carvacrol and *p-*cymene, respectively. However, when the two compounds were added to the juice together, *E. coli *could not be counted or detected after 1 day of exposure to the treatment (Figure 2 and Table 2). Plating out on selective agars with or without a thin layer of TSA gave very similar results to those shown in Figure 2 and so these counts are not illustrated.

It was noted that the addition of 0.5 mM carvacrol and 0.25 mM p-cymene, alone or in combination, imparted a very slight "spicy" aroma to the treated juices.

Throughout this study, the pH of the juices remained essentially unchanged at around 3.20 ± 0.06, irrespective of the type of treatment or storage temperature.

## Discussion

Freshly-pressed apple juice prepared from healthy fruit can be expected to contain around 3–5 log CFU/ml of viable microorganisms, of which the majority are yeasts [23]. The results in this study were consistent with this expectation, as shown in Figures 1 and 2. The pH of the juices used in this study was at the lower end (3.20 ± 0.06) of the expected pH range (2.9–4.5) for fresh apple juice [23].

Despite the low pH, *E. coli *O157:H7 survived in raw apple juice for 3 days at 25°C and nearly 3 weeks (19 d) at 4°C. These findings agree with those reported elsewhere [23-28]. The acidic nature of apple juice does not ensure its safety as *E. coli *O157:H7 may survive for extended periods of time, especially at chill temperatures.

The antimicrobial properties of carvacrol and similar phenolic compounds from the essential oils of herbs and spices have been reported previously against individual microorganisms, tested *in vitro *[12,20,29-32]. The application of carvacrol in the preservation of some foods such as rice and fresh-cut fruit has also been reported [18,22]. However, this is the first report of the successful application of relatively low doses (0.25–1.25 mM) of carvacrol and *p-*cymene against *E. coli *O157:H7 in the presence of the mixed microflora of fruit juice. The results demonstrate that the addition of 1.25 mM carvacrol or *p-*cymene to apple juice inactivated *E. coli *O157:H7 within less than 1 day from about 4 log CFU/ml to levels that were undetectable using conventional microbiological techniques (Figure 1). Furthermore, once inactivated, *E. coli *remained undetectable for the duration of the trial (20 days; Table 1). In addition, the results obtained using the thin agar layer method for resuscitating injured organisms were no different from those using selective media alone, suggesting that the cellular injury caused by the phenolic compounds was irreversible. The substantial reduction in the number of days (from 19 to 1) that *E. coli *O157:H7 was able to survive in apple juice when treated with 1.25 mM carvacrol or *p-*cymene and stored at chill temperatures represents an opportunity to improve the safety of un-pasteurised fruit juices.

In addition to their antibacterial activity, carvacrol and *p-*cymene were also biocidal against the yeast flora naturally present in the apple juice (Figure 1). However, 1.25 mM *p-*cymene was not as effective as the same concentration of carvacrol in eliminating the yeast population at ambient temperature. Those yeasts surviving the treatment with *p-*cymene recovered and eventually reached the same viable numbers as in the untreated control. By contrast, the presence of either phenolic compound at 1.25 mM resulted in similar, gradual die-off of the yeast population at chill temperatures. The results confirm the broad antimicrobial spectrum of carvacrol but also suggest that yeasts may be somewhat less sensitive to phenolic compounds than bacteria. The reduction in numbers of spoilage yeasts would clearly benefit both the manufacturer and consumer by extending the shelf-life of the product.

When carvacrol and *p-*cymene were added to apple juice at 0.5 and 0.25 mM, respectively, *E. coli *O157:H7 and the yeast population were reduced but to a different extent than was observed at the higher concentrations of 1.25 mM. The shapes of the inactivation curves for the total viable numbers (Figure 2) would suggest that there may have been some synergism between carvacrol and *p-*cymene but the results for *E. coli *(Figure 2 and Table 2) suggest that the effect may have been additive. In the presence of 0.5 mM carvacrol and 0.25 mM *p-*cymene used alone, *E. coli *was not detectable for 13 and 6 d longer than in the control but in the presence of both compounds used in combination, the organism was not detectable for 18 d longer. Therefore, it is not possible to conclude unequivocally whether the effect of adding the two substances was synergistic or merely additive.

It has been reported previously that carvacrol was more effective in reducing the viable count of the natural microflora on kiwifruit (pH 3.2–3.6) than on honeydew melon (pH 5.4–5.5) [22]. At low pH, the hydrophobicity of essential oil components increases, enabling them to partition more easily into the lipids of the cell membrane of bacteria.

It is known that direct plating on selective media following exposure to physical or chemical stresses can lead to gross underestimation of viable counts by as much as 3–4 log CFU ml^-1 ^[33,34]. Although a thin agar layer method [35] and enrichment techniques [36] were used in this study to allow some resuscitation of the inoculated pathogen, it is possible that the results shown in Figures 1 and 2 represent an underestimate of numbers. Furthermore, it is possible that a larger sample volume (25 ml instead of 2.5 ml) in the pre-enrichment step would have allowed the resuscitation and recovery of an even greater number of *E. coli *from the control juices.

In the present study, ethanol was used as a solvent for the preparation of stock solutions of carvacrol and cymene. Whilst the final concentrations of ethanol in the juices were below the tolerance limit previously reported for *E. coli *[37], it is possible that the low levels of ethanol present (0.95, 1.9 and 4.75% in the presence of 0.25, 0.5 and 1.25 mM carvacrol/cymene, respectively) may have potentiated the biocidal action of the phenolic compounds. This possibility would need experimental confirmation in future work.

## Conclusion

When inoculated into un-pasteurised apple juice, *E. coli *O157:H7 survived for up to 3 and 19 days at 4° and 25°C, respectively. At 1.25 mM and at both storage temperatures, carvacrol and *p-*cymene reduced the numbers of *E. coli *O157:H7 to undetectable levels within 1–2 days. The effective concentrations of carvacrol could be reduced even further by combining it at 0.5 mM with *p-*cymene at 0.25 mM. Carvacrol and *p-*cymene were biocidal against both spoilage yeasts and *E. coli *O157:H7 thereby increasing the shelf-life and improving the safety of un-pasteurised apple juice, particularly when stored at chill temperatures.

## Methods

### Materials

Freshly pressed, unclarified, raw juice from a mixture of Bramley and Cox apples was obtained directly from a manufacturer in Suffolk, England. The pH of the juice was 3.20 ± 0.06 on arrival at the laboratory. All microbiological media and diluents were from Oxoid (Basingstoke, UK) and all chemicals from Sigma Chemicals Co. Ltd. (Poole, Dorset, UK) unless otherwise indicated.

Un-pasteurised apple juice is often referred to as "cider" in the USA. This should not be confused with UK cider, which is a fermented alcoholic beverage. In this paper, the term "apple juice" refers strictly to the raw, unprocessed, unfermented juice of the apple.

### Microorganisms and their cultivation

Bacteria, yeasts and moulds were routinely cultivated on Plate Count Agar (PCA) and Malt Extract Agar (MEA). PCA plates were incubated at 30°C for 2–3 days whilst MEA plates were incubated at 25°C for 3–5 days. The attenuated strain of *Escherichia coli *O157:H7 (Dr. P. McClure, Unilever Research, Colworth House, Bedford, UK) was maintained in Brain Heart Infusion (BHI) broth and agar and incubated overnight at 37°C. For challenge experiments, fresh overnight cultures of *E. coli *O157:H7 in BHI broth were washed three times and re-suspended in sterile saline solution. Viable counts of the washed cell suspensions were determined by serial dilution (1:10) in Maximal Recovery Diluent (MRD) and spread-plating 0.1 ml of the suspensions (in duplicate) on Tryptone Soya Agar (TSA) and incubating the plates at 37°C for 24 h.

### Treatment of apple juice with carvacrol and cymene

Containers (200 ml capacity) of apple juice (100 ml) were prepared in duplicate for each treatment and stored at 4°C and 25°C. Stock solutions (25 mM) of carvacrol and cymene were prepared in 95% ethanol and added to the apple juice to give final concentrations of 0.25, 0.5 and/or 1.25 mM. The juice was inoculated with the washed suspension (prepared as described above) of *E. coli *O157:H7 to give an approximate count of 10^4 ^CFU/ml. Control juices inoculated with *E. coli *O157:H7 but containing no antimicrobials, as well as absolute controls containing no antimicrobials or *E. coli *O157:H7 were also prepared.

Samples (10 ml) were taken periodically for up to 20 days from each container for microbiological analysis and pH determination. Serial (1:10) dilutions were prepared in sterile MRD. Total viable numbers were determined by pour-plating (1.0 ml) on PCA. Yeasts and moulds were enumerated by spread-plating (0.1 ml) on Tryptone Glucose Yeast Extract Agar (TGYEA) supplemented with chloramphenicol (0.1 mg/ml). PCA and TGYEA plates were incubated at 30°C for 2–3 days. Single samples were removed from each duplicate batch of juice periodically during incubation for viable counting and plating out in triplicate; therefore, mean counts for each time point were calculated from six replicate determinations.

Pre-enrichment and enrichment steps were used to detect low levels of *E. coli *O157:H7 in the apple juice following treatment with carvacrol and/or cymene. An aliquot of 2.5 ml of juice was added to 22.5 ml BPW and incubated at 37°C for 24 h (in the case of the un-inoculated control, 25 ml apple juice was added to 225 ml BPW). Following incubation, an aliquot (1 ml) was dispensed into 10 ml EC broth (reduced bile salts supplemented with novobiocin) and incubated at 37°C for 24 h. A loopful of the EC broth was streaked onto Sorbitol MacConkey (SMAC) Agar or onto CHROMagar (M-Tech Diagnostic Ltd., EE-220-Trial; both agars supplemented with cefixime and potassium-tellurite) and incubated at 37°C for 24 h.

For enumeration of injured cells of *E. coli *O157:H7, the Thin Agar Layer method (TAL) was used [35]. Spread plates were prepared on selective agars (CT-SMAC agar or CHROMagar supplemented with cefixime and potassium-tellurite) and on selective agars overlaid with TSA agar. The number of injured cells was calculated by subtracting the viable count on selective agar alone from the viable count obtained on the overlaid agar.

## Authors' contributions

GK participated in the design of the study and carried out all the experimental work.

SR conceived the study and participated in its design and coordination.

GK and SR drafted the manuscript jointly.

## Figures and Tables

**Table 1:** Presence/Absence of *E. coli *in apple juice stored at 25°C (A) and 4°C (B). Results (in duplicate) were obtained by enrichment of samples from the experiment illustrated in Figure 1.

A										
Antimicrobial treatment	Time (days)									
	
	0	1	2	3	4	7	12	15	19	20

Control, no additions	-	-	-	-	-	-	-	-	-	-
Control, *E. coli *O157:H7 inoculated	+	+	+	+	-	-	-	-	-	-
Carvacrol, 1.25 mM	+	±	-	-	-	-	-	-	-	-
Cymene, 1.25 mM	+	±	±	-	-	-	-	-	-	-
B										

Antimicrobial treatment	Time (days)									
	
	0	1	2	3	4	7	12	15	19	20

Control, no additions	-	-	-	-	-	-	-	-	-	-
Control, *E. coli *O157:H7 inoculated	+	+	+	+	+	+	+	+	+	-
Carvacrol 1.25 mM	+	-	-	-	-	-	-	-	-	-
Cymene 1.25 mM	+	-	-	-	-	-	-	-	-	-

**Table 2:** Presence/Absence of *E. coli *in apple juice stored at 4°C. Results (in duplicate) were obtained by enrichment of samples from the experiment illustrated in Figure 2.

**Antimicrobial treatment**	**Time (days)**																				
	
	0	1	2	3	4	5	6	7	8	9	10	11	12	13	14	15	16	17	18	19	20
**Control, no additions**	-	-	-	-	-	-	-	-	-	-	-	-	-	-	-	-	-	-	-	-	-
**Control, E. coli O157:H7 inoculated**	+	+	+	+	+	+	+	+	+	+	+	+	+	+	+	+	+	+	+	+	-
**Carvacrol, 0.5 mM**	+	+	+	+	+	+	+	±	-	-	-	-	-	-	-	-	-	-	-	-	-
**Cymene, 0.25 mM**	+	+	+	+	+	+	+	+	+	+	±	+	±	±	+	-	-	-	-	-	-
**Carvacrol (0.5 mM) plus cymene (0.25 mM)**	+	+	-	-	-	-	-	-	-	-	-	-	-	-	-	-	-	-	-	-	-
